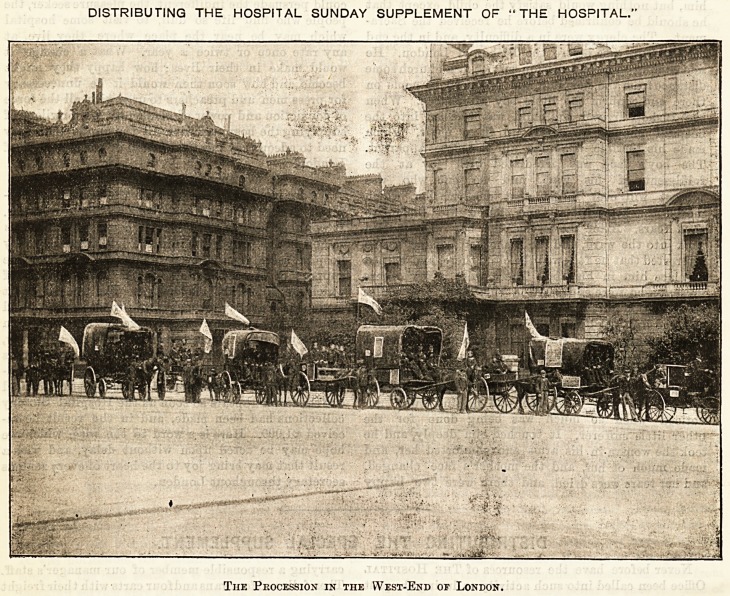# Distributing the Special Supplement

**Published:** 1895-06-22

**Authors:** 


### DISTRIBUTING THE SPECIAL SUPPLEMENT.

Never before have the resources of The Hospital Office been called into such activity as during the past week—that preceding Hospital Sunday. It seemed a sufficiently large task to supply the ministers of the larger churches with copies of our special supplement for distribution amongst their congregations upon the previous Sunday. But the striking procession which passed down the Strand at ten o'clock on Thursday and Friday mornings before Hospital Sunday, bearing tens of thousands of these supplements, showed what hearty goodwill, hard work, and generosity could accomplish in an endeavour that every house in well-to-do London in 1895 should possess strikingly illustrated facts and figures showing the work and needs of the hospitals, the subject of nuiversal appeal throughout the metropolis on Sunday.

That the appeal should really reach the hands for whom it was intended, a carefully thought out and complete plan of distribution was organised, the expense of which, together with the production of the many thousands of copies of the supplement necessary, was supplied by a few generous citizens.

Two days were devoted to delivering the supplement throughout the wealthier portions of the West and South-west of London. In the morning an orderly and striking procession started from Messrs. George Smith and Sons, who were charged with the no light task of addressing the envelopes containing the appeal. Heading the procession came the brougham carrying a responsible member of our manager's staff. Then followed four vans and four carts with their freight of neatly uniformed commissionaires and boys charged with the distribution. Over the vans floated white flags with scarlet lettering, " Special Hospital Sunday Appeal." Scarlet posters and other fixtures rendered the procession striking and effective. The distributing army wore special badges, and were systematically directed and supervised, a commanding officer being in charge of each detachment, whilst the manager's brougham did good work in reporting progress all along the line. Every delivery of one of the supplements necessitated ringing a door-bell and the attendant waiting for response, and as fifty envelopes were distributed by each man or boy per hour, those responsible have every reason to be satisfied at the despatch with which the work was effected. After driving to the scene of distribution selected for each day, the word of command was given for vans and carts to disperse to their area of work, all meeting again at some given hour to parade home, the most popular route possible being chosen, so that what the ear would not hear at least the eye must see. The procession created no little curiosity and interest, and was a most happy method of attracting the attention of Londoners to the forthcoming Hospital Sunday. The procession assembled on Friday afternoon in Porchester Terrace, where an effective photograph was taken, which we produce on the next page.

### DISTRIBUTING THE HOSPITAL SUNDAY SUPPLEMENT OF "THE HOSPITAL."

The Procession in the West-End of London.

There were many amusing incidents in connection with the distribution, and not least amongst these have been the contents of our letter-bag. Distributing agencies throughout London have evidently been struck with the success of our efforts, and thinking we may make circularising our speciality, the applications to assist us have been numerous. Amongst others, the Salvation Army offer to lend their services. We are very content with the success of our efforts. We consider ourselves fortunate in our choice of a distributing corps, all of whom worked well, two complaints only having reached us. One was from the employer of an indignant housemaid, whose head came in contact with a flying "Supplement" delivered by a youthful commissionaire whose spirits got the better of him on completing two days' hard work, and who was not sufficiently impressed even by his connection with a Hospital Sunday Fund movement. The other came from a rich gentleman who remonstrated with us for our effectual method of securing delivery promptly, as he said the envelope was made so important it might have been sent after him *anywhere*. Considering the hundreds of pounds expended on this endeavour to induce the well-to-do to give, and the amount of personal service freely offered, we were not as penitent at risking these few pence of our rich correspondent as, we fear, he expected! We had an amusing complaint from a school boy, who objected to our artist's superscription on the coins given in the Supplement. He is very much afraid they will corrupt the Latin of the West-end; whilst his brother finds a weak point in the article, "Raising the Wind," where a hint to cricket clubs is given. His cricket club, we learn, is in want of funds itself, and therefore those of our readers who are interested in hospital finance should give to this cricket club, since he argues, the more athletes the fewer patients. We are interested in the prosperity of both the members and the funds of cricket clubs, which we hope may grow and flourish, and thus benefit our hospitals directly and indirectly, but we fear we cannot start another special appeal just yet. We have received many striking proofs of the interest and goodwill our procession, and the cause it embodied, has met with in our metropolis; and we hope our readers in the provinces will accord us interest and sympathy also, for in the provincial towns a hearty interest in local charities is more easily evinced than in London, where, however, we hope, on this occasion at any rate, we have focussed the attention of London upon Hospital Sunday.

## Figures and Tables

**Figure f1:**